# Health Tract, No. 229

**Published:** 1865

**Authors:** 


### CHURCH VENTILATION.

MANY persons have gone to church, taken cold, gone home, and died in a few days, from sitting in an ill-warmed or ill-ventilated church, arising from the inattention or ignorance of sextons or indifference of church-officers; hence tens of thousands are interested to the extent of life and death in the perusal of these few lines. Perhaps three persons out of four, who attend divine service on the Sabbath-day, are conscious, within two minutes after taking their seats, that they have been in a hurry; that both mind and body have been more or less in a turmoil; they have been hurried in getting to church in time; the result is, they are overheated, that is, the body is in a state of warmth considerably above what is natural, and if in this condition they sit still, even for ten minutes, in an atmosphere cooler than that of out-doors in summer, or below sixty degrees at any time, a cold is the result, slight or more severe, according to the vigor and age of the individual. What would give but a trifling cold to a person in robust health, would induce inflammation of the lungs, called by physicians pneumonia, in an old person, or any one of infirm health. Many a person has taken cold and died of pneumonia in three or four days, although in perfect health previously, by sitting a few minutes in a fireless room in winter-time. The danger is still greater if the room has been closed for several days; this is specially applicable to houses of worship. Within a few minutes after the benediction, at the close of the Sabbath services, the house is shut up, doors, windows, and all; the atmosphere of the building has been saturated with the breath of the worshipers; as it becomes gradually cooler, this dampness condenses and falls toward the floor, so does the carbonic acid gas, which is what becomes so unpleasantly perceptible on entering a sleeping-chamber after a morning-walk, and there is experienced a sepulchral dampness and closeness enough to chill any one on first entering the church, after having been closed several days. We once knew a gentleman, who was something of an invalid, to take a chill and die in a short time, from entering a warehouse in December, which had been closed for a week or two.

The practical conclusion is, that every church ought to have the windows and doors open for several hours, including the middle of the day, before it is opened for service. In cold weather, preparatory to the Sabbath service, this ventilation should be secured on Friday, and early on Saturday mornings fires should be built and steadily kept up, day and night, until the Sabbath services are concluded. A thermometer should be kept hanging about five feet from the floor, near the center of the building, and the mercury should be kept at about sixty-five or seventy degrees in fire-time of year—better seventy than under sixty-five.

